# Pseudopaline-mediated zinc uptake by *Pseudomonas aeruginosa* drives clinically relevant phenotypes and infection outcomes

**DOI:** 10.1128/iai.00453-25

**Published:** 2026-01-14

**Authors:** Lola Bosc, Thomas Sécher, Geneviève Ball, Deborah Le Pennec, Mathilde Tribout, Moly Ba, Yingjie Bai, Laurent Ouerdane, Pascal Arnoux, Yann Denis, Xiaoguang Lei, Christophe Bordi, Nathalie Heuzé-Vourc’h, Susanne Häussler, Nicolas Oswaldo Gomez, Romé Voulhoux

**Affiliations:** 1 Laboratoire de Chimie Bactérienne UMR7283, Centre National de la Recherche Scientifique, Aix-Marseille Université 27051, Marseille, France; 2 Respiratory Disease Research Centre U1100, INSERM, University of Tours 27092, Tours, France; 3 Laboratoire d’Ingénierie des Systèmes Macromoléculaires UMR725, Aix-Marseille Université, Centre National de la Recherche Scientifique 128791, Marseille, France; 4 Department of Chemical Biology, College of Chemistry and Molecular Engineering, Peking University 598979, Beijing, People's Republic of China; 5 Centre National de la Recherche Scientifique, Institut des sciences analytiques et de physico-chimie pour l'environnement et les matériaux UMR5254, Université de Pau 131730, Pau, France; 6 Institut de Biosciences et Biotechnologies d'Aix-Marseille UMR7265, Commissariat à l’Energie Atomique, Aix-Marseille Université 128791, Saint-Paul-lez Durance, France; 7 Institut de Microbiologie de la Méditerranée Centre National de la Recherche Scientifique, Aix-Marseille Université 128791, Marseille, France; 8 Department of Molecular Bacteriology, Helmholtz Centre for Infection Research GmbH, Braunschweig, Germany; 9 Center of Clinical and Experimental Infection Research (TWINCORE), a joint venture of the Hannover Medical School and the Helmholtz Center for Infection Research, Institute for Molecular Bacteriology, Hannover, Germany; 10 Department of Clinical Microbiology, Copenhagen University Hospital—Rigshospitalet 683301, Copenhagen, Denmark; Tsinghua University, Beijing, China

**Keywords:** *Pseudomonas aeruginosa*, pseudopaline, metallophore, metal uptake, biofilm, pathogenicity, virulence

## Abstract

Biological metals are vital trace elements required by metalloproteins, which are involved in virtually every cellular, structural, and catalytic function of the bacterial cell. Bacterial pathogenesis involves a tug-of-war between the host’s nutritional immunity sequestering essential metals and the invading pathogens that deploy adapted high-metal affinity uptake strategies, such as metallophores, in order to efficiently circumvent these defense mechanisms. Pseudopaline is a metallophore produced and secreted by *Pseudomonas aeruginosa* to acquire zinc when the bioavailability of this metal is severely restricted, as in the presence of a strong metal chelator such as EDTA, or during infections when the nutritional immunity of the host is active. We show that when facing strong metal chelation, the general Znu zinc uptake pathway becomes ineffective and only the pseudopaline pathway is capable of supplying the bacteria with the necessary zinc to maintain their growth, establishing that the pseudopaline pathway is the last-resort pathway for the bacteria to acquire zinc under such restricted growth conditions. Based on this statement, the present study explores the pleiotropic role of pseudopaline-mediated zinc acquisition on clinically relevant phenotypes such as biofilm formation and associated antibiotic tolerance, as well as its capacity to determine infection outcomes using cell-culture and murine models. The expression of pseudopaline-dependent phenotypes in such a diversity of biological contexts demonstrates the essentiality of this specific metal uptake system for *P. aeruginosa* pathogenicity during infection. We therefore identify this machinery as a promising therapeutic target for *P. aeruginosa* infections.

## INTRODUCTION

Biological metal ions are essential across all kingdoms of life (1). Their unique chemical features provide structural and catalytic properties to a wide range of metalloproteins, broadly distributed in living organisms (2). Zinc is, after iron, the second most abundant metal in living cells, and zinc-binding proteins represent 6% to 9% of the prokaryotic and eukaryotic proteomes, respectively (3, 4). In bacteria, zinc metalloproteins are involved in several functions essential not only to growth, such as central metabolism or DNA replication and repair, but also to pathogenicity and virulence alike virulence factors, antibiotic resistance, motility, extracellular protease activities, and modulation of the immune response (5, 6).

Biological metals are inorganic resources that need to be acquired by bacteria from the environment where they are often rare, either because of low concentration, restricted bioavailability, or competition between the inhabitants of the same ecological niche. In the specific biotope that the host-pathogen interface represents, both protagonists have developed competing strategies for either sequestering or acquiring the much-coveted metal. Hence, the host synthesizes high-affinity chelators, which withhold essential metals through the process referred to as “nutritional immunity” (7). Conversely, pathogens have evolved powerful metal uptake systems involving high-affinity secreted molecules called metallophores (8) that often determine their ability to survive during infections.

Thus, invading bacterial pathogens face a particularly dire restriction of metals during human infection, and specifically zinc due to its sequestration by the host zinc-binding protein calprotectin, broadly conserved in mammals (7, 9–13). During human infection, and in cystic fibrosis (CF) patients in particular, the levels of calprotectin in bronchoalveolar lavage fluid (BAL) and serum are elevated and used as a biomarker of diseases correlating with exacerbation (14–16). To responsively overcome the growth-limiting zinc deficiency in tissues, imposed by nutritional immunity, the human pathogen, *Pseudomonas aeruginosa,* has developed effective zinc import mechanisms judiciously de-repressed under zinc deficiency by the Zur regulator (formerly known as np20). Zur is a cytoplasmic repressor protein that prevents gene transcription by binding to the *zur* box (GTTATagtATAtC) of its promoter region, and the magnitude of repression is proportional to the intracellular zinc concentration (17). Thus, the lower the intracellular zinc concentration, the less active Zur is, and the greater the transcription of the initially repressed gene (18). *P. aeruginosa* is therefore able to sense zinc scarcity in its environment in order to react by gradually de-repressing the Zur regulon, comprising, among others, the Znu and Cnt (also called Zrm) zinc import mechanisms (12, 19). The Znu pathway, widely distributed in bacteria, recognizes and imports free zinc in the cytoplasm via the periplasmic substrate-binding protein ZnuA and the inner membrane importer ZnuBC (19, 20). In order to acquire zinc when it is not free but sequestered by a powerful chelator, such as EDTA or calprotectin, *P. aeruginosa* relies on the dedicated Cnt pathway involving the high zinc affinity zincophore pseudopaline (21–24). We summarize in Fig. S1 the known steps of pseudopaline biosynthesis and trafficking through the bacterial membrane.

Numerous transcriptomic studies have reported a systematic upregulation of the *cnt* operon under the infection conditions of CF patients or in medium reproducing these conditions (12, 25–31). Moreover, the *cnt* genes are particularly conserved in *P. aeruginosa* clinical isolates (32), and the promoter of these genes is the target of mutations during adaptation to CF patients, which often increase the expression of the *cnt* genes in evolved strains (33). Together with the requirement of the Cnt pathway for *P. aeruginosa* growth in airway mucus secretion (34), these data indicate that the Cnt pathway is not only induced but also critical for *P. aeruginosa* to successfully establish an infection.

We have developed a minimal growth medium called minimal chelated medium (MCM) consisting of minimal succinate (MS) medium supplemented with 100 µM of the divalent metal chelator EDTA (22). We show that under such severe zinc sequestering conditions, the alternative Znu zinc uptake pathway becomes ineffective, rendering the pseudopaline mandatory for efficient zinc acquisition from the medium since a *P. aeruginosa* Δ*cntL* strain—with a functional Znu pathway—is unable to maintain a normal intracellular zinc homeostasis. In line with this statement, we demonstrate in this study the pleiotropic role played by pseudopaline in *P. aeruginosa* infections to overcome metal scarcity. We first establish its central role to secure the intracellular zinc concentration required for optimal planktonic growth of the pathogen in MCM medium, independently of the Znu machinery. Moreover, we show that in such zinc-limiting conditions, the Cnt pathway is necessary to establish a mature biofilm which provides protection from antibiotic exposure. We further explored the role of pseudopaline in environments encountered during *P. aeruginosa* infection of the host. Specifically, during bacterial interaction with macrophages, we demonstrate its role in adherence, phagocytosis, intracellular survival, and induction of immune responses. Additional *in vivo* experiments in a mouse model of *P. aeruginosa* lung infection confirmed the requirement for this zincophore to colonize the lung and develop a full virulent phenotype. Altogether, our data revealed that the pseudopaline metallophore produced and secreted by *P. aeruginosa* gives it the exceptional ability to maintain a proper intracellular zinc homeostasis under conditions of extreme metal deficiency, conditions encountered on several occasions during the nutritional immunity imposed by the host during infection.

## RESULTS

### The Cnt pathway is the last resort pathway safekeeping zinc homeostasis in severe metal-sequestered growth condition

**Fig 1:**
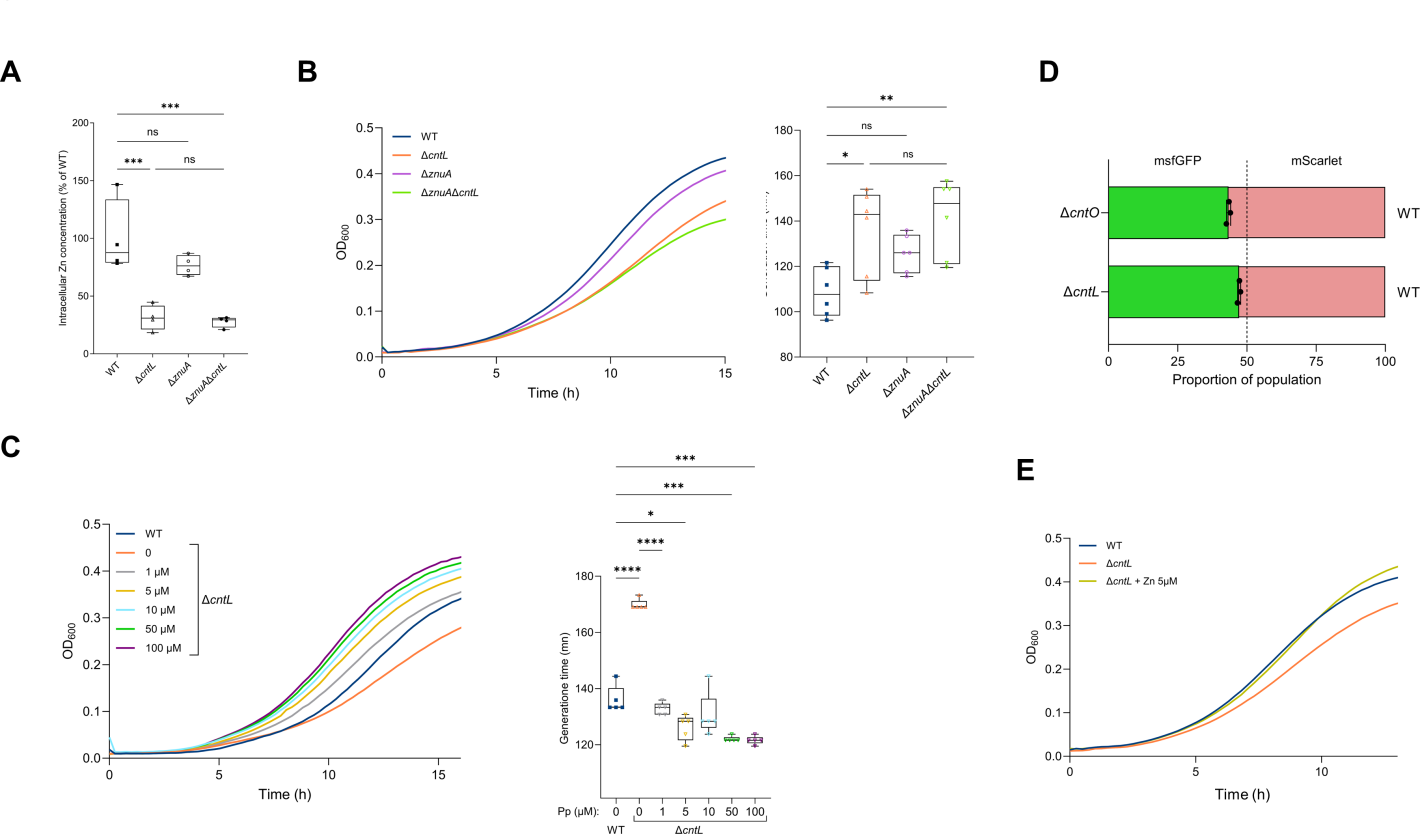
The pseudopaline pathway, but not the Znu one, is required for optimal intracellular zinc homeostasis and planktonic growth in MCM. (**A**) Intracellular zinc concentrations and (**B**) growth curves and corresponding generation times of PA14 WT, Δ*cntL*, Δ*znuA,* and Δ*znuA*/Δ*cntL* strains in MCM. (**C**) Impact of externally added pseudopaline on cell growth of WT and Δ*cntL* strains, supplemented or not with various concentrations (from 1 to 100 µM) of exogenous pseudopaline, grown in MCM. Complete growth curve (left) as well as generation times (right) are presented. (**D**) Proportion of populations after a 10-h co-culture in MCM of PA14 WT-mScarlet with Δ*cntL-msfGFP* or Δ*cntO-msfGFP* evaluated by flow cytometry (*n* = 3, 100,000 events per biological replicate). (**E**) Impact of externally added zinc on cell growth of PA14 WT and Δ*cntL* strains, supplemented or not with 5 µM of exogenous zinc (Zn) and grown in MCM. Error bars, mean ± standard deviation (sd) of at least three independent biological replicates. ns, not significant; ****, ***, **, and * correspond to *P* < 0.0001; *P* < 0.001; *P* < 0.01 and *P* < 0.05, respectively (one-way ANOVA, Tukey post-hoc test).

We previously showed that under conditions of low zinc bioavailability due to extracellular sequestration by EDTA, as the one created in MCM (22), the normal intracellular zinc level and optimal planktonic bacterial growth are ensured by pseudopaline since in its absence (in the Δ*cntL* strain), the intracellular zinc content decreases, and bacterial growth is impaired compared with the parental wild type (WT) strain (Fig. 1A and B and as previously shown [21]). Considering the described role of the Znu machinery as a zinc importer (19, 20), we further question whether this pathway supports or at least contributes to the lower growth of the Δ*cntL* strain in MCM. The similar MCM intracellular zinc concentration and generation times, reported Fig. 1A and B between the WT and the single Δ*znuA* strain on the one hand and the single Δ*cntL* and the double Δ*cntL*/Δ*znuA* strains on the other hand indicate that this is not the case. These data demonstrate that under conditions of extremely low zinc availability, such as those created in MCM, the Znu import pathway is no longer operational, and the necessary zinc for the optimal growth of *P. aeruginosa* is imported by the zincophore pseudopaline. We next confirmed that the growth defect of the Δ*cntL* strain depends exclusively on the presence of pseudopaline as supplementation by exogenous addition of 1μM of synthetic pseudopaline restores the growth defect of the Δ*cntL* strain (Fig. 1C, gray curve). This synthetic supplementation of the Δ*cntL* strain with externally acquired pseudopaline, in agreement with its *cis-*complementation by the WT *cntL* gene previously demonstrated (21), not only excludes any polar effect of the deletion but also confirms that the mode of action of pseudopaline involves an extracellular acquisition stage. This agrees with the three-step cycle of synthesis, secretion, and recovery that we previously proposed for the pseudopaline-dependent zinc uptake pathway (21). Additional experiments performed with different concentrations of externally added synthetic pseudopaline indicate that optimal WT growth is already achieved at 1 µM (Fig. 1C), revealing that in the metal-limiting conditions encountered in MCM, the natural extracellular concentration of pseudopaline does not exceed 1 µM. Moreover, the observation that the addition of higher concentrations of pseudopaline leads to better growth than that observed in the WT strain also indicates that zinc recovered by pseudopaline under these conditions is the limiting factor for growth in MCM. As shown for other metallophores, once secreted in the media, these molecules become “*common goods”* and benefit the overall bacterial population, leading to division-of-labor strategies (35). To explore this possibility, we grew co-cultures of either Δ*cntL* or Δ*cntO* strains with their parental strain, expressing either msfGFP or mScarlet, respectively, and measured the relative proportion of each strain after a 10-h culture in MCM. At the end of this experiment, we recovered a similar proportion of cells of WT and Δ*cntL* during their co-culture, while a lower proportion of Δ*cntO* cells was recovered in co-culture with WT (Fig. 1D). This indicates that the relative fitness of a Δ*cntO* strain is impaired compared with its Δ*cntL* counterpart. This can be explained by the fact that, once secreted, pseudopaline is a shared resource that benefits a non-producer, but not a strain which cannot produce its cognate receptor.

Despite the ability of pseudopaline to bind other divalent metals (24), we also show that the growth defect of the Δ*cntL* strain in MCM is effectively due to zinc deficiency since it is specifically restored by extracellular zinc supplementation (Fig. 1E) and not by other divalent metals (Fig. S2). Furthermore, we recorded the transcriptome of the Δ*cntL* mutant and WT strains during growth in MCM and observed only minor differences (Fig. S3A; Table S1). Among the differentially regulated transcripts, the only significant functional enrichment based on gene ontology (GO) terms identified a minor downregulation of C4-dicarboxylate transport and terpene catabolic processes (Fig. S3B). This information suggests that the molecular mechanisms underlying the growth defect we observe in metal-scarce conditions could be post-transcriptional, because of the absence of Zn cofactors necessary to proteins involved in central processes.

Altogether, our data based on planktonic growth indicate that in MCM, zinc is scarce and only the secreted pseudopaline can import enough of this metal to guarantee its intracellular levels, needed to feed the zinc proteome and ensure optimal planktonic bacterial growth.

### The pseudopaline pathway is required for biofilm formation and antimicrobial resistance in MCM

**Fig 2:**
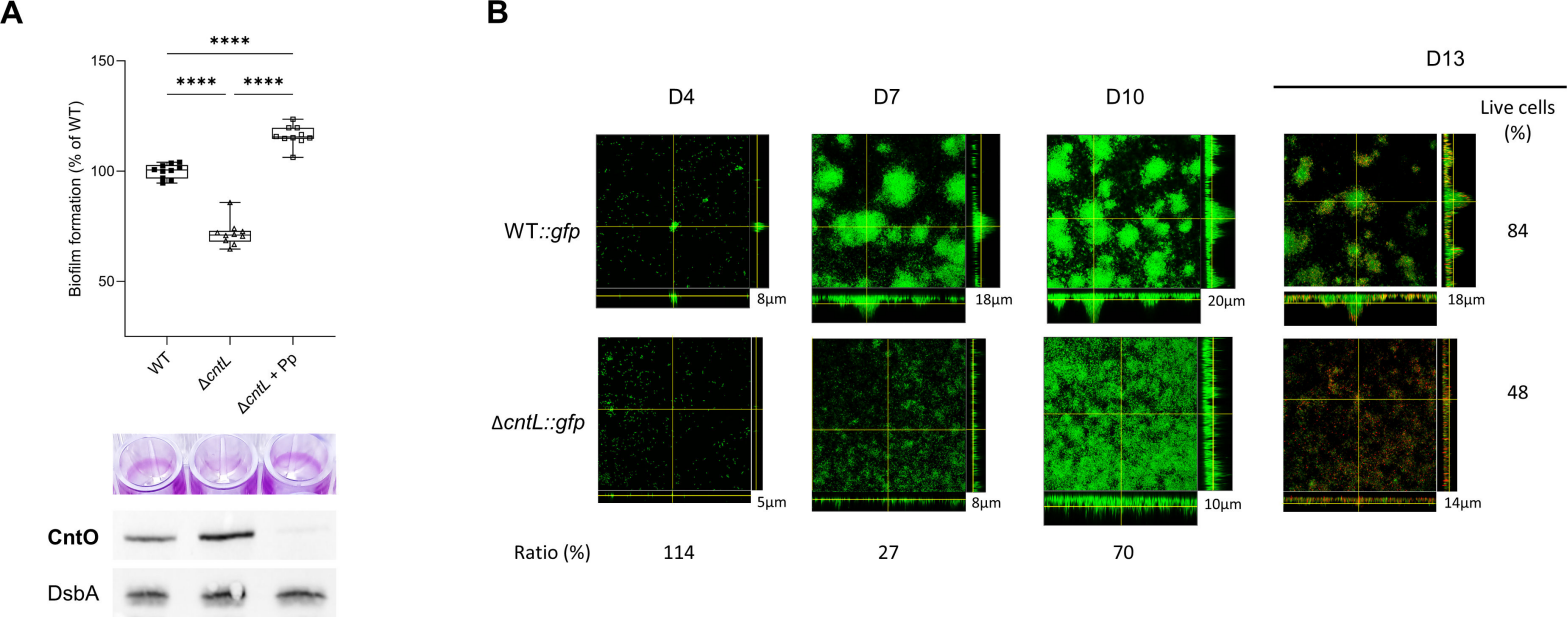
The pseudopaline pathway is required for biofilm formation and antimicrobial resistance in MCM. Impact of pseudopaline on biofilm formation of (**A**) PA14 WT and Δ*cntL* strains or (**B**) GFP-labeled PA14 WT (WT::*gfp*) and Δ*cntL* (Δ*cntL::gfp*) strains, supplemented or not with exogenous pseudopaline (Pp) at 100 µM and grown in MCM. (**A**) Biofilm formation was measured by quantifying bacterial mass adhering to the walls of a microtiter plate well as described in the Materials and Methods section. A representative illustration of crystal violet rings obtained with the different strains during the biofilm formation assay is presented in the middle panel. CntO and housekeeping DsbA productions in the different protein samples visualized by immunoblot experiments are presented in the lower panel. (**B**) Qualitative biofilm formation was monitored at days 4 (D4), 7 (D7), and 10 (D10) post-incubation. Stacked confocal scanning laser microscopy images of biofilms and corresponding extracted z images and their respective xy and xz planes, as well as their thickness (in µm) are presented revealing their classic pilar- and mushroom-shaped architecture. The ratios (in %) indicate the average biomass of the Δ*cntL* strain (PA14Δ*cntL::gfp*) compared with the WT (PA14wt::*gfp*) strains at the three different days of incubation. Antimicrobial tolerance of PA14wt::*gfp* and PA14Δ*cntL::gfp* strains from 11 days aged biofilms exposed to 20 μg/mL of tobramycin for 24 h and observed at day 13 (D13) after 24 h of additional growth. Cells with a compromised membrane that are dead or dying stain red with propidium iodide, whereas cells with an intact membrane stain green with GFP fluorescence. The live cells (in %) indicate the biomass of the live cells compared with the total live and dead cells obtained from two independent experiments. The experiment was repeated twice, independently, and the images presented correspond to the typical results observed in both experiments. Error bars, mean ± standard deviation (SD) of at least three independent biological replicates. **** correspond to *P* < 0.0001 (one-way ANOVA, Tukey post-hoc test).

In order to evaluate the implication of pseudopaline in clinically relevant conditions, we explored zinc acquisition in biofilm formation, the most common lifestyle reported during infections (36). This multicellular organization protects the bacterial community from external onslaughts, such as phagocytosis or killing by antibiotics and allows for establishing chronic recalcitrant infections. We compared biofilm formation of WT and Δ*cntL* strains grown in MCM using several quantitative and qualitative approaches. We first examined biofilm formation in 24-well polystyrene plates after 24 h of static growth in MCM at 30°C. The Δ*cntL* strain, unable to produce pseudopaline, shows a significant 30% reduction of crystal-violet stained biofilm compared with the WT (Fig. 2A). Adding synthetic exogenous pseudopaline to the Δ*cntL* strain restores biofilm formation, confirming that, alike in planktonic growth, this biofilm phenotype is also exclusively due to the absence of pseudopaline.

To further explore the biofilm phenotype related to intracellular zinc deficiency, and since intracellular zinc concentration cannot be precisely measured in 24-well polystyrene plates, we indirectly evaluated intracellular zinc level by monitoring CntO production as a reporter of intracellular zinc concentration. Planktonic transcriptional data, together with metal and protein level measurements, all presented Fig. S4, indeed, revealed a direct relationship between intracellular zinc concentration and the amounts of CntO protein produced (Fig. S4A) or the corresponding transcripts (Fig. S4B). The use of this read-out in biofilm samples first confirmed the zinc default in the Δ*cntL* strain as well as the import of zinc by the exogenously added pseudopaline, respectively illustrated by higher and strong reduction of CntO protein detection compared to the WT level (Fig. 2A, bottom). The restoration of biofilm in the mutant by 100 µM pseudopaline to a level significantly higher than that observed in the WT also supports that, similar to the planktonic growth (Fig. 1C), pseudopaline-dependent zinc import is also the limiting factor for biofilm formation. This result shows that in *P. aeruginosa* biofilms grown under zinc-limited conditions, the zinc homeostasis required for its optimal formation is exclusively ensured by pseudopaline.

In order to ensure that this biofilm defect in the absence of pseudopaline is not only due to the growth defect (Fig. 1A) and to assess the qualitative impact of the absence of pseudopaline on biofilm formation in MCM, we used confocal microscopy to follow its formation and organization in flow cell experiments using genetically engineered strains expressing constitutively the green fluorescent protein (GFP) in *cis* at a neutral site on the chromosome. We imaged biofilms formed by WT and Δ*cntL* strains after 4, 7, and 10 days of incubation in a flow cell chamber. Biomass ratios and thickness measurements presented in Fig. 2B show significant reductions in cell number and thickness of the biofilm in the absence of pseudopaline after 7 and 10 days of incubation. From a qualitative point of view, the Δ*cntL* strain formed biofilms with an impaired morphology compared with the parental WT strain, unable to form the classic pilar- and mushroom-shaped architecture (Fig. 2B). We wondered if these morphological alterations were indicative of variation in antibiotic tolerance, a property described for *P. aeruginosa* biofilms (37). To do so, we measured the capacity of these biofilms to withstand the offense of tobramycin treatment, an aminoglycoside antibiotic commonly used by inhalation with CF patients suffering from chronic infection (38). D11 biofilms were treated for 24 h with 20 µg/mL of the aminoglycoside. The live (GFP-green) and dead (propidium iodide-red) dyes applied at D13 highlight a significantly higher sensitivity to tobramycin for the Δ*cntL* strain compared with the WT (Fig. 2B right panels). This observation was not reported in planktonic growth in MCM where WT and Δ*cntL* strains were both sensitive from 4 µg/mL (Fig. S5). Together, we infer that the absence of pseudopaline not only delays biofilm formation but also significantly impairs its structural properties and its ability to confer tolerance to an antibiotic. The negative effect of the absence of pseudopaline on the volume and quality of the biofilm in metal-scarce conditions is most likely attributable to the impact of the reduced intracellular zinc level—reported by the higher amount of CntO detected (Fig. 2A lower panel)—on the proper function of bacterial zinc-dependent metalloproteins involved in this process (39).

The pseudopaline-dependent growth and biofilm phenotypes observed in this study (Fig. 1 and 2) highlight the broad and exclusive importance of this metallophore in environments where zinc is scarce and chelated, such as the one created in MCM or reported during infections where nutritional immunity leads to serious metal deficiencies in general, and zinc in particular (7, 9, 12, 13).

### The impact of pseudopaline on the sequence of events during macrophage infection suggests multiple roles for this molecule

**Fig 3:**
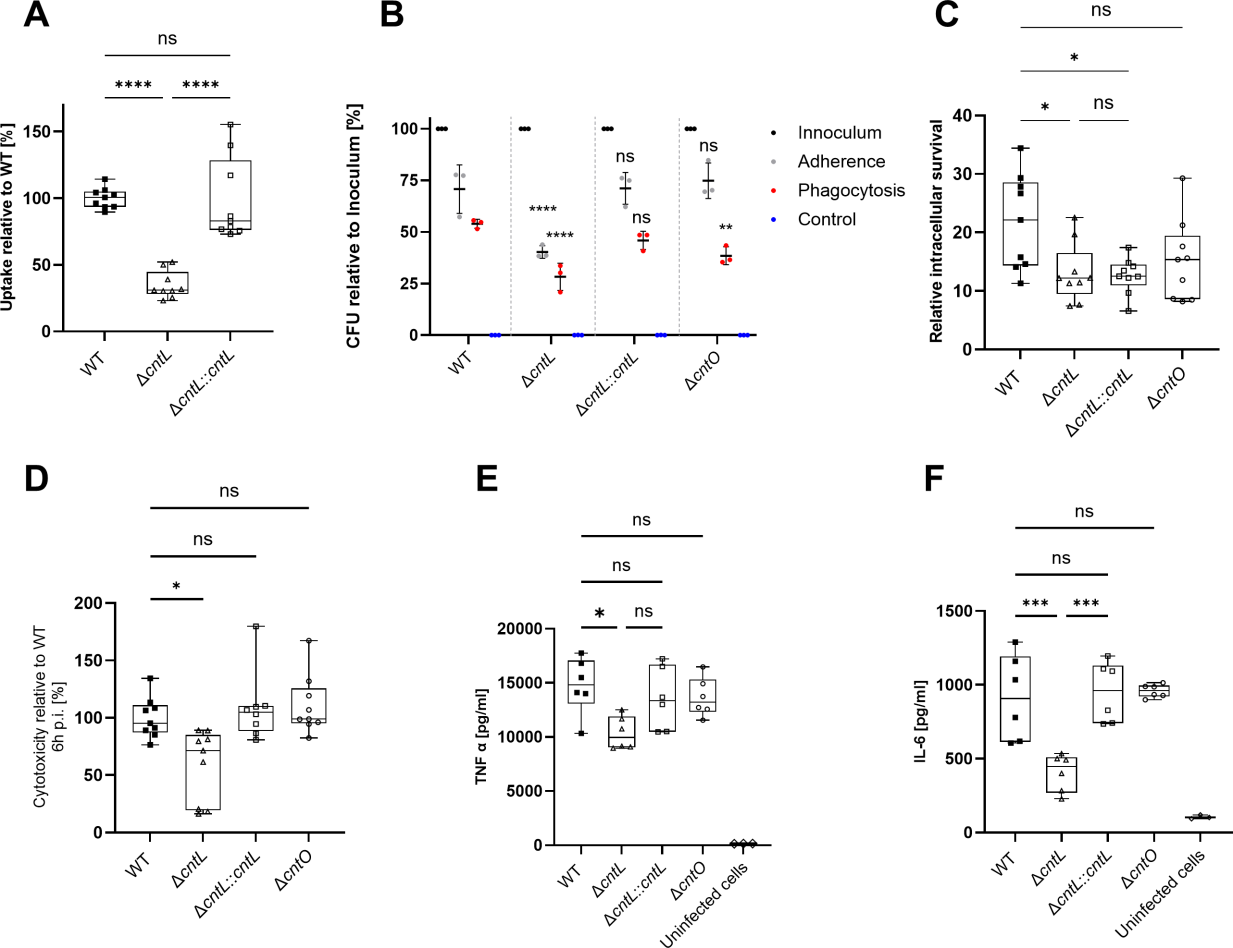
The pseudopaline pathway is sequentially required at different stages of macrophage infection by *P. aeruginosa*. Strains PA14 WT, Δ*cntL*, Δ*cntO,* and Δ*cntL cis-*complemented (Δ*cntL::cntL*) strains, precultured in MCM, were compared in different macrophage infection assays. (**A**) Phagocytic uptake into RAW 264.7 cells. The results of three independent experiments with three biological replicates are presented. **** correspond to *P* < 0.0001 (one-way ANOVA, Tukey post-hoc test). (**B**) Adherence measurement to RAW 264.7 cells, following treatment of cells with cytochalasin D to stop phagocytosis (gray). The inoculum (black) was set as 100% for each strain, and the uptake without cytochalasin D (corresponding to phagocytosis) was also run as an internal control (red), as well as a negative control with cytochalasin and gentamicin in which no CFU were recovered (blue). Mean ± standard deviation of three biological replicates is shown. Statistical differences indicated on the figure correspond to the value obtained with WT strain. ** and **** correspond to *P* < 0.01 and *P* < 0.0001, respectively (two-way ANOVA, Dunnett post-hoc test). (**C**) Bacterial survival in RAW 264.7 macrophages. Macrophages were infected by *P. aeruginosa* strains and treated with gentamicin 1 hpi. The ratio of CFU recovered 6 hpi was normalized to CFU recovered 2 hpi and expressed as percentage. The results of three independent experiments with three biological replicates are depicted. * and ** correspond to *P* < 0.0332 and *P* < 0.0021, respectively (one-way ANOVA, Tukey post-hoc test). (**D**) Cytotoxicity on RAW 264.7 macrophages. Cells were infected at an MOI of 1 after culture in MCM and treated with gentamicin 1 hpi. Supernatant was sampled 6 hpi, and lactate dehydrogenase (LDH) activity was measured by a colorimetric assay. Values are normalized to LDH activity upon infection with PA14 WT strain, expressed as percentage. The results of three independent experiments with three biological replicates are depicted. ** correspond to *P* < 0.0021 (one-way ANOVA, Tukey post-hoc test). Quantification of tumor necrosis factor α (TNF-α) (**E**) and interleukin 6 (IL-6) (**F**) produced 6 hpi by RAW 264.7 macrophages infected by *P. aeruginosa* strains at MOI 1 after growth in MCM and treated with gentamycin 1 hpi. The results of two independent experiments with three biological replicates are depicted. * and ** correspond to *P* < 0.05 and *P* < 0.001, respectively (one-way ANOVA, Tukey post-hoc test). ns, not significant.

To further explore the role of pseudopaline at different steps of a host-cell infection process, we resorted to a RAW 264.7 macrophage-like murine cells model (40). We started by quantifying phagocytic uptake through a gentamycin-protection assay and observed that the Δ*cntL* strain pre-cultured in MCM is significantly less phagocytosed than the parental WT strain, a phenotype mostly recovered in the complemented strain (Fig. 3A). We hypothesized that this reduced phagocytic uptake could be due to a reduced adherence of the Δ*cntL* strain to the cell membrane of macrophages. To address this hypothesis, we blocked phagocytosis by adding 10 µM cytochalasin D to the culture medium before infection and determined adherent bacterial cells. We observed that the Δ*cntL* strain indeed showed a decreased adherence to the macrophages (Fig. 3B), thus explaining, at least in part, the reduced phagocytic uptake. Interestingly, we also observe that a Δ*cntO* strain is not affected in adherence and presents a significantly reduced phagocytosis, albeit to a lower extent than the Δ*cntL* mutant.

Following phagocytosis, macrophages employ several strategies to clear bacterial cells, including both nutrient starvation and metal ion toxicity (41). We evaluated the role of pseudopaline on bacterial survival in the phagosome by measuring the number of CFU recovered 6 h post-infection (hpi). To account for the decreased phagocytosis of the Δ*cntL* strain compared with the WT strain, we normalized the survival to the number of CFU phagocyted 2 hpi. Under these phagocytosis-independent conditions, the Δ*cntL* strain shows a significantly reduced survival than the WT (Fig. 3C). Surprisingly, while all our phenotypes insofar were complemented in the Δ*cntL::cntL* strain, this was not the case for bacterial survival in macrophages, although the complemented strain produces enough pseudopaline to satisfy the operational requirements of this uptake pathway and supply enough zinc to maintain metal homeostasis. We also previously observed a significant reduction in the production of pseudopaline in this complementing strain (22), which may result from the transcriptional reduction of its corresponding *cntL* biosynthesis gene, observed in the present study (Fig. S6). To assess whether pseudopaline could have an alternative role as a buffering agent against metal intoxication in the phagolysosome, we quantified the survival of a Δ*cntO* strain in the same model. In the absence of its outer membrane receptor, pseudopaline is produced but cannot be recovered by the bacteria, hereby partially phenocopying the zinc deficiency of our Δ*cntL* strain, but maintaining the role of the secreted pseudopaline as a buffering agent. We observed that the relative intracellular survival of the Δ*cntO* mutant had no statistical difference with the parental WT strain (Fig. 3C). Altogether, this information suggests that, unlike all the other phenotypes described in this manuscript, the impact of pseudopaline on intracellular survival could arise from a dual use of this molecule, and in this specific case results from a buffering strategy to counter metal ion toxicity. Of note and considering the ability of pseudopaline to bind other divalent metals (24), we believe that this role could extend beyond its zinc binding properties to other transition metals and therefore extend the spectrum of its buffering property.

Additionally, we also observe that, during macrophage infection, a Δ*cntL* strain is less cytotoxic (Fig. 3D) and less immunogenic than the parental one. This is shown by the decreased production of TNF-α (Fig. 3E) and IL6 (Fig. 3F) produced by macrophages when infected by the Δ*cntL* strain. Owing to our knowledge of the reduced Zn-metallome of this strain, the missing cofactor of several proteins involved in these processes is impacting these properly-complemented phenotypes. Interestingly, we observe no decreased cytotoxicity and immunogenicity of a Δ*cntO* strain, suggesting that pseudopaline could have a role *per se*, that we cannot fully decouple in our experiments from its function as a metal-shuttle.

These results demonstrate the multiple roles of pseudopaline on the sequence of events during macrophage infection. They also validate that the nutritional zinc deficiency imposed on the pathogen by the macrophage triggers the induction of the pseudopaline pathway. Pseudopaline acts as a zinc-cargo able to displace this atom from otherwise bio-unavailable source to feed the operating requirement of various zinc-binding proteins, which are particularly relevant during the interaction with host cells. Pseudopaline can also be used as a buffering agent against bursts of ions, as is the case in the phagosome. Both roles are key during the interaction with the host and underline the critical role of pseudopaline in establishing pathogenicity.

### Pseudopaline plays a critical role during *P. aeruginosa* pulmonary mouse infection

**Fig 4:**
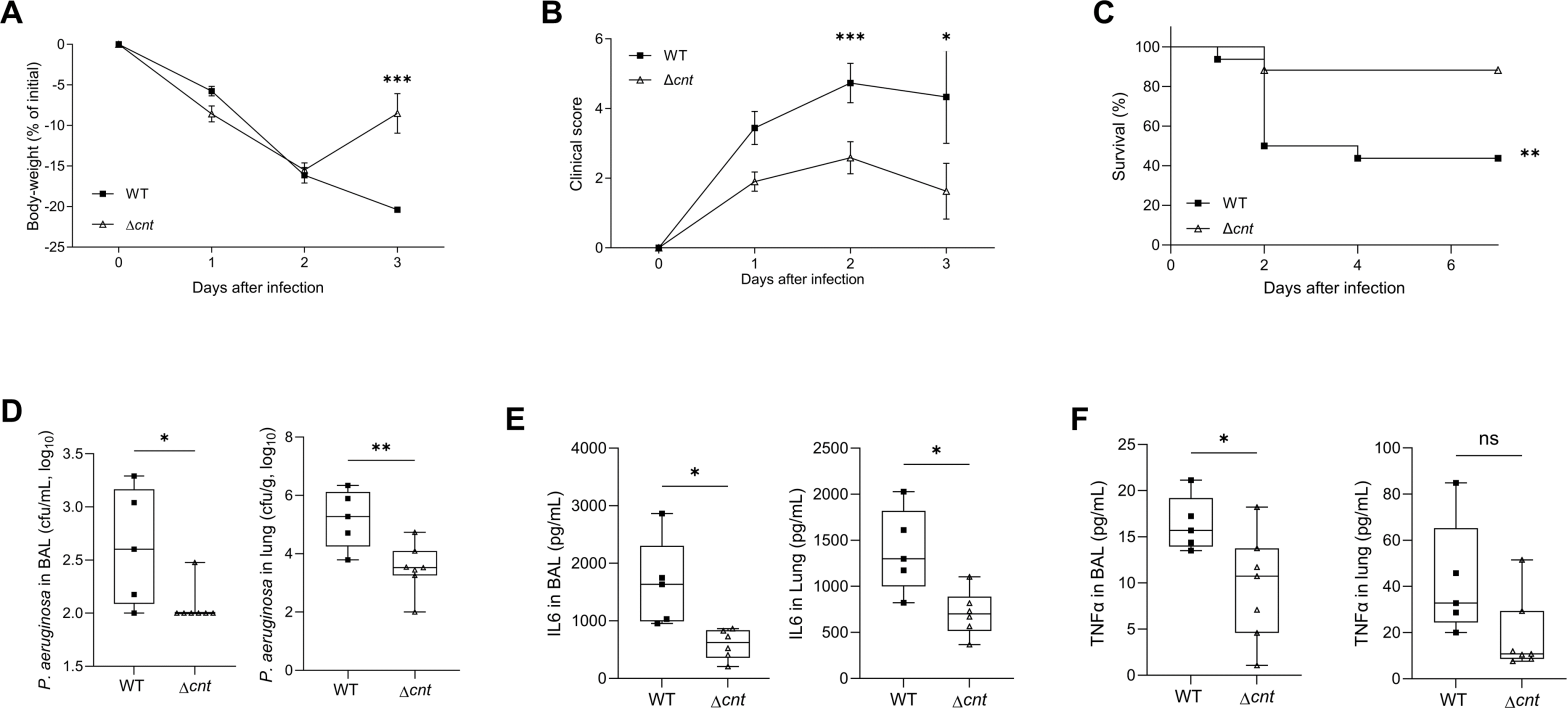
The pseudopaline pathway is key to *P. aeruginosa* pulmonary infection. Mice were infected intratracheally with PA14 WT and Δ*cnt* strains precultured in MCM. Animal weight (**A**), clinical score (**B**), and survival (**C**) were recorded throughout the experiment as described in the Materials and Methods section. (**D**) In a separate experiment, mice were sacrificed at 2 days post-infection, and the number of PA14 in the BAL and lungs was determined. The amount of (**E**) IL-6 and (**F**) TNF-α was measured in the BAL and lungs. Each result corresponds to at least three independent experiments (*n* = 17/group) for panels **A–C** and one experiment (*n* = 5–7/group) of panels **D and E**. ns, not significant; *, **, and *** correspond to *P* < 0.05, *P* < 0.01, and *P* < 0.001, respectively (one-way ANOVA, Tukey post-hoc test).

To further extend our cell culture-based findings to an *in vivo* animal model, we used a well-established mouse pulmonary infection model (42). Mice were infected by pulmonary instillation of MCM-grown WT and a mutant strain devoid of the whole *cntOLMI* operon, referred to as Δ*cnt*. Several markers, including body weight, clinical score, and survival, were recorded during the infection. While all mice infected with the WT strain suffered from severe conditions characterized by continuous body-weight loss (Fig. 4A), increased clinical score (Fig. 4B), and > 50% death 4 days post-infection (Fig. 4C), the mice infected with the Δ*cnt* strain exhibited significantly milder evidence of susceptibility, including regain of body-weight loss 2 days post-infection (Fig. 4A), less severe clinical symptoms (Fig. 4B), and almost full survival after 7 days post-infection (Fig. 4C). To gain insight into the mechanisms that contributed to the lower virulence associated with the Δ*cnt* mutant, we analyzed the host’s antibacterial responses 48 hpi. Mice infected with the WT strain showed significantly higher bacterial load in bronchoalveolar fluid (BAL) and lungs as compared with *cnt* mutant-infected animals (Fig. 4D). Given that pneumonia is intimately associated with lung inflammation, we evaluated the production of IL6 and TNF-α, two prototype pro-inflammatory markers, in the BAL and in the lungs (Fig. 4E and F). As observed with macrophage infections, the present analysis revealed that IL6 and TNF-α were reduced in the animals infected by the *cnt* mutant as compared to the WT. Altogether, these data confirm the attenuation of virulence of the pseudopaline-deficient *P. aeruginosa* strain in a mouse model of lung infection.

## DISCUSSION

Zinc fulfills multiple functions in living cells, and its intracellular concentration must therefore constantly be finely regulated, whatever the environment. In *P. aeruginosa,* at least three different levels of zinc import processes are operating, depending on its scarcity. When zinc is abundant in the milieu, its entry is mediated by passive diffusion across the outer membrane followed by low-affinity import mechanisms to reach the cytoplasm, as already reported for *E. coli* (43). When zinc starts to run out, the Zur regulon is consequently induced, leading to the activation of the dedicated Znu and Cnt high-affinity zinc uptake systems. Under moderate zinc starvation conditions and as soon as free zinc is still available in sufficient amounts, zinc uptake is mainly mediated by the dedicated Znu uptake pathway since intracellular level of zinc is significantly affected in the corresponding *znuA* mutant (19, 20, 23). This observation also means that the Cnt pseudopaline-mediated zinc import pathway cannot fully complement the Znu pathway in such moderate zinc scarcity. This could be explained by a lower induction of this pathway at this level of zinc deficiency, in agreement with the levels of *cntO* induction proportional to zinc deficiency reported in Fig. S4. Nevertheless, while the widely distributed Znu system ensures high-affinity free zinc uptake under conditions of moderate deficiency, we are demonstrating in this study that the pseudopaline-dependent Cnt zinc uptake pathway of *P. aeruginosa* confers this bacterium the ability to deal with highly pronounced zinc deficiency conditions. A context imposed by a high level of metal chelator in MCM or by the host’s nutritional immunity during infection, where the Znu pathway, specialized in the import of free zinc, is no longer suited. In line with this context-dependent hypothesis, the significant contribution of the Znu pathway in the bacterial zinc homeostasis reported in previous studies (19, 20, 23) is most likely due to the lower degree of metal chelation achieved with either citrate or only 10 µM EDTA or TPEN, whereas the MCM used in the present study contains 10 times more EDTA. This explanation is supported by planktonic growth examination of *P. aeruginosa* strains in various metal chelation conditions. Using optimal growth in MCM under various EDTA concentrations as a read-out for the necessity of both Cnt and Znu pathways, we are showing Fig. S7 that in the severe zinc scarcity of the MCM medium (100 µM EDTA), the Znu pathway is unable to rescue the CntL negative growth phenotype, while it is able to do it when a less severe zinc scarcity (10 µM EDTA) is applied. The ZnuA-dependent growth phenotype observed in the Δ*cntL* strain at 10 µM EDTA but not at 100 µM agrees with an involvement of this pathway only at moderate zinc starvation and also confirms the effective inactivation of the *znuA* gene in the corresponding mutant strain.

Given that zinc import is essential for the bacterium and that the *cnt/znu* double mutant still grows, albeit poorly, in MCM (Fig. 1), it is highly likely that this bacterium possesses additional and yet-to-be-identified mechanisms for zinc import. Recent studies propose that the pyochelin siderophore could have an alternative function and play a role in zinc uptake (44). Several other candidate machineries, belonging to the Zur regulon, have also been proposed by Pederick and colleagues (19). These include the two TonB-dependent receptors PA2911 and PA1922, characteristic of this metal import systems and which could, with their respective genetically encoded partners subjected to the same Zur regulation, form additional zinc import machineries (45). Unlike other bacterial species, *P. aeruginosa* therefore seems to be particularly well equipped to acquire zinc in all circumstances, which could give it a singular advantage in the course of the infection (46).

We used synthetic pseudopaline to perform supplementation experiments and confirmed that the pseudopaline zinc uptake pathway is a cyclic process where the metallophore is first synthesized and secreted by the bacterium to then be regained by the bacterium in its holo form loaded with zinc. This observation could already have been deduced from two independent studies demonstrating first that pseudopaline is produced by CntL and then secreted into the supernatant (22), and second, that the addition of supernatants harvested from the WT rescued the growth of a strain lacking *cntL* but did not rescue the growth of a Δ*cntO* strain (23). In agreement, planktonic co-culture experiments in metal-scarce conditions show that the relative fitness of a Δ*cntL* strain is comparable to the WT strain, while a Δ*cntO* strain is more affected (Fig. 1D). These also reveal that once secreted, pseudopaline is a “common good” that benefits bacteria in the same niche able to acquire it, which could lead to mutualist relationships in polymicrobial context.

Despite indirect evidence suggesting a release of the metal from the pseudopaline in the periplasm (21), the fate of the pseudopaline-metal complex once imported by CntO remains elusive. As the *znuA* mutant does not show any growth defect in MCM (Fig. 1), we are, however ruling out the existence of an interplay between the two pathways and the import in the cytoplasm of the pseudopaline-imported zinc by the Znu machinery. Thus, the molecular journey of pseudopaline after crossing the outer membrane involves either a metal release in the periplasm or a translocation of holo-pseudopaline through the inner membrane. In both cases, either the metal alone or the holo-pseudopaline needs a dedicated inner membrane transporter that remains to be discovered.

In addition to its general role in bacterial planktonic growth, zinc also promotes biofilm formation in *P. aeruginosa* (39). It is therefore not surprising to find that zinc uptake systems become important for biofilm formation in zinc-limited environments, such as the one encountered during lung infection. In line with this, Mastropasqua and colleagues report significant alteration of biofilm formation and its associated traits such as swimming and swarming motility in the double Δ*znuA/*Δ*cntO* strain compared with the WT grown in moderate VB-MM zinc-deplete medium (28). We also previously showed that under more severe zinc depletion—growth in MCM—where zinc uptake is only mediated by the Cnt pathway and its metallophore pseudopaline, the Δ*cntL* strain presents a reduced quantitative amount of biofilm (21). In the present study, we are expanding the pseudopaline-mediated CntL biofilm phenotype in MCM to fine qualitative alteration and the consequential antibiotic tolerance. These results demonstrate the importance of pseudopaline in biofilm formation under the conditions of high zinc deficiency encountered during infection, where this metallophore becomes necessary for proper zinc import.

The protective role of pseudopaline at different infection stages of the macrophage observed in this study may counteract several metal-related processes set up by the macrophage to fight the pathogen. Much of the research on nutritional immunity has indeed shown a general sequestration by the host of metal ions to limit microbial growth, a process also observed in activated macrophages to limit zinc availability to intracellular pathogens (47), which the pseudopaline-mediated zinc import pathway logically mitigates. It is now also clear that additional and complementary processes, centered on TLR-regulated metal ion trafficking, induce metal intoxication of the pathogen inside the macrophagic vacuole by modulating macrophage-mediated metal ion transport systems (6). Our data unravel a protective role of pseudopaline inside the vacuole, which is explained by a dual extracellular role of pseudopaline allowing to buffer large burst of metal, as already reported for copper by the mycobacterial metallothionein MymT (48). Such extracellular buffering function of pseudopaline in the macrophage vacuole is supported by the lack of complementation of the intracellular survival phenotype, shown in Fig. 3C. The genetic engineering of this strain, relying on the insertion of a *cnt* promoter fusion with the *cntL* gene at a neutral chromosomal site, leads to a reduced production of pseudopaline in the supernatant (21). We identified herein that this is a direct consequence of a sixfold transcriptional down-regulation compared with its parental strain (Fig. S6). It is remarkable that the reduced production of pseudopaline in our complemented strain seems to concern only the intracellular survival phenotype (Fig. 3C). We rationalized that this Cnt phenotype is the only one for which an optimal level of extracellular pseudopaline is necessary to counteract, through a buffering activity, intoxication by sudden divalent-metals burst considering its high affinity for most divalent metal (24). Thus, a reduced production of pseudopaline decreases bacterial survival in the phagolysosome. This dual use of pseudopaline as a metal-buffering agent is also supported by the improved survival of a Δ*cntO* strain, not restricted in pseudopaline production and secretion but unable to import it (Fig. 3C). Interestingly, the fact that the Δ*cntO* strain is unaffected in its cytotoxicity (Fig. 3D) and immunogenicity (Fig. 3E and F) suggests that pseudopaline could also have a role on the host, regardless of the roles we demonstrated as an uptake and buffering bacterial agent. Alternatively, we previously described that the absence of the encoded TBDT has an intermediate phenotype between WT and a mutant unable to produce pseudopaline regarding its metal uptake in MS (21). Therefore, we cannot rule out a threshold effect, during which the Δ*cntO* mutant has still enough Zn-uptake to ensure function of proteins involved in these phenotypes. Further studies are needed to conclude if pseudopaline has a molecular role *per se* on the host cells.

Induction of the *cnt* pathway in the macrophage vacuole is demonstrated by the phenotype reported in its absence (Fig. 3C) as well as by the DualSeq detection of specific *cntO* and *cntL* transcripts in the macrophage (40). Such induction of the *cnt* operon implies zinc deficiency in this compartment, where zinc bursts have, however, been reported during bacterial infections (49, 50). This apparent contradiction can be explained by the temporal separation of the two events during macrophage infection to which the pathogen has adapted through various subversion mechanisms (51). The buffering role of pseudopaline, as proposed in this study, could be added to this list.

The pseudopaline-deficient phenotype in the mouse infection model reported in Fig. 4 indicates an important role for the pseudopaline Cnt pathway in *P. aeruginosa* pulmonary pathogenesis. While using an intraperitoneal sepsis mouse model, Mastropasqua and colleagues report a loss of fitness of the Δ*cntO* strain compared with the WT that also supports our findings (23). More generally, the numerous mice or human transcriptome data showing a systematic up-regulation of the *cnt* genes under similar pulmonary infection conditions therefore extend the role played by the pseudopaline to pulmonary infections in general (26, 28, 31). The pseudopaline pathway is therefore important for *P. aeruginosa* survival in a mammalian host when it must cope with severe metal deficiencies, such as those encountered in pulmonary infections and possibly due to sequestration by calprotectin (52).

Results presented in this study revealed that under metal scarce and chelating conditions, such as the one encountered during pulmonary infections, the Cnt pseudopaline zinc uptake pathway is the last-resort pathway allowing sufficient zinc uptake into the bacterium to preserve the proper intracellular zinc concentration necessary to maintain the pool of zinc metalloproteins fully functional. We are reporting in this study several clinically relevant phenotypes affected by zinc privation due to the absence of pseudopaline, of which the underlying molecular mechanisms are likely post-transcriptional and can be explained by the presence of a large number of zinc-metalloproteins whose function depends on the supply of cofactors. Several zinc metalloproteins have previously been shown to be important for cellular functions including transcription, translation, motility, or protease activity.

The identification of the essential and multifactorial role of pseudopaline in *Pseudomonas* infections paves the way for the development of new antibacterials against *P. aeruginosa* infections. Such molecules hold promise to act as synergetic pathoblockers potentially able to resensitize established biofilms to current and available antibiotic therapies. In addition, the observation that the Cnt pathway is at play during infection and the fact that pseudopaline is taken up in the periplasm by its specific CntO receptor offers the perspective of using pseudopaline as a scaffold for Trojan horse-inspired chemistry, as previously tried with other metallophores (53).

Nicotianamine-like metallophores structurally similar to pseudopaline have also been reported in other bacteria (54). Interestingly, our finding that pseudopaline is the last-resort mechanism to overcome the severe zinc scarcity encountered during infection is consistent with the similar function described for the opine-type metallophore staphylopine, which enables *Staphylococcus aureus* to compete with the host’s calprotectin to overcome nutritional immunity during infection (55). Together with our findings, these data strongly suggest that this family of metallophores may play an important role in the pathogenesis of several microbes. Finally, and more broadly, the fact that *P. aeruginosa* is found in non-pathogenic environments and that numerous non-pathogenic environmental bacteria appear to encode for nicotianamine-like metallophores (54, 56) extends their range of action and competence well beyond the host-pathogen relationship.

## MATERIALS AND METHODS

### Bacterial strains and preculture or planktonic growth conditions

Bacterial strains used in this study are listed in Table 1. All *P. aeruginosa* strains used in this study are derivatives of the parental PA14 strain. In macrophage infection experiments, the absence of *cntL* in the PA14Δ*cntL* strain was complemented by *cis* chromosomal expression of the *cntL* gene under the *cnt* operon promoter, inserted at the *attB* site of the *P. aeruginosa* chromosome (22). For co-cultures, the parental WT strain as well as the Δ*cntL* and Δ*cntO* strains were genetically engineered to express a specific fluorophore *in cis* under control of the strong, constitutive, P_EM7_ promoter. Unless otherwise specified, precultures and planktonic growths of all *P. aeruginosa* strains were performed in minimal succinate (MS) medium (per liter: 6 g K_2_HPO_4_, 3 g KH_2_PO_4_, 1 g (NH_4_)_2_SO_4_, 0.2 g MgSO_4_, 4 g succinic acid, and 3.1 g NaOH, pH 7.0) and minimal chelated medium MCM (MS medium supplemented with 100 µM EDTA), respectively, both at 37°C with horizontal shaking in sterile disposable polycarbonate Erlenmeyer flasks or 96-well plates in order to avoid any metal contamination and monitored by OD_600_ measurements.

**TABLE 1:** Bacterial strains used in this study

*P. aeruginosa* strain	Description	Reference
PA14 WT	WT strain	(57)
PA14 WT::*gfp*	WT PA14 strain harboring the *gfp* gene under constitutive promoter at *att* site	(This study)
PA14∆*cntL*	Δ*cntL* deletion strain	(22)
PA14∆*cntL::gfp*	PA14∆*cntL* strain harboring the *gfp* gene under constitutive promoter at *att* site	(This study)
PA14∆*cntL::cntL*	PA14∆*cntL* harboring a wild type copy of *cntL* gene at *att* site	(22)
PA14∆*cntO*	Δ*cntO* deletion strain	(22)
PA14 ∆*znuA*	Δ*znuA* deletion strain	(This study)
PA14 ∆*cntL*∆*znuA*	Δ*znuA* Δ*cntL* double-deletion strain	(This study)
PA14∆*cnt*	PA14 strain deleted for the complete *cntOLMI* operon.	(21)
PA14 WT *attB::P_EM7_-mScarlet*	PA14 WT strain bearing a *P_EM7_-mScarlet* reporter at *attB* site	(This study)
PA14 ∆*cntL attB::P_EM7_-msfGFP*	PA14 ∆*cntL* strain bearing a *P_EM7_-msfGFP* reporter at *attB* site	(This study)
PA14 ∆*cntO attB::P_EM7_-msfGFP*	PA14 ∆*cntO* strain bearing a *P_EM7_-msfGFP* reporter at *attB* site	(This study)

### Construction of *znuA* and *znuA-cntL* deletion mutant strains of *P. aeruginosa*

Two DNA fragments corresponding to upstream And downstream regions of the *znuA* gene were amplified from PA14 chromosomal DNA with PCR primers Mut-*znuA*-1/Mut-*znuA*-2 and Mut-*znuA*-3/Mut-*znuA*-4 (Table 2). Upstream and downstream regions were ligated by overlapping PCR and cloned into linearized pKNG101 (58) by the SLIC method (59). The resulting constructs were transformed into *E. coli* CC118λ*pir* and introduced into *P. aeruginosa* PA14 by conjugation. The strains in which the chromosomal integration event occurred were selected on *Pseudomonas* isolation agar plates supplemented with 2 mg/mL streptomycin. Excision of the plasmid, resulting in the deletion of the chromosomal target gene, was performed after selection on Luria-Bertani (LB) plates containing 6% sucrose. Sucrose-resistant and streptomycin-sensitive clones were confirmed to be deleted for the *znuA* gene by PCR analysis using Mut-*znuA*-5/Mut-*znuA*-6. The Δ*znuA* Δ*cntL* strain was constructed by knocking out *znuA* in the Δ*cntL* strain.

**TABLE 2:** Primers used in this study

Name	Sequence (5′−3′)
Mut *znuA*-1	CAGGTCGACGGATCCCCGGGATCAGGCGTCCTTCTGGTC
Mut *znuA*-2	CACGGGTTTTCACACGGCGGCACT
Mut *znuA*-3	AGTGCCGCCGTGTGAAAACCCGTGACT
Mut *znuA*-4	TATGCATCCGCGGGCCCGGGTTTCTCGAGGATGTTGTCCA
Mut *znuA*-5	CGATCAGGGTGACGATCTG
Mut *znuA*-6	TGACGGTGAAGCACATCTTG
qRT*cntO*For1	CGCCATTTCTCGTTGAACTC
qRT*cntO*Rev1	ACGAACTGATCCTGCGTAGC
QRT*cntL*For1	ATTTGTGCTGCCTGGACATC
QRT*cntL*Rev1	AGCGAAGCGATCAGGAAAT
*uvrD*1	CACGCCTCGCCCTACAGC
*uvrD*2	GGATCTGGAAGTTCTGCTCAGC
*16S* For	CAGCTCGTGTCGTGAGATGT
*16S* Rev	GATCCGGACTACGATCGGTT
NOG648	ATGCGATATCTGTTGACAATTAATCATCGGCATAGTATATCGG
NOG649	GCATCTCGAGCTGGATTCTCACCAATAAAAAACGCCC

### Construction of PA14 strains constitutively expressing fluorophores

DNA fragments corresponding to fusions of the strong constitutive promoter PEM7 and the coding sequence of fluorophores msfGFP and mScarlet were amplified from pSEVA2313 variants (60) using primers NOG648/NOG649. The PCR product was purified, digested with EcoRV/XhoI restriction enzymes (ThermoFisher), and cloned into linearized mini-CTX1 plasmid by ligation. The constructs were transformed into *E. coli* SM10λ*pir,* sequenced, and introduced into *P. aeruginosa* PA14 by conjugation. The recombinant clones containing the mini-CTX1 inserted at the *attB* locus on the *P. aeruginosa* genome were selected on tetracycline-containing PIA. Excision of the sequence located between the FRT sites was achieved by expressing Flp recombinase from the pFLP2 plasmid, which was introduced by electroporation. Finally, selection for pFLP2 deficient strains was done after selection on LB plates containing 6% sucrose. Colonies that became sucrose-resistant and carbenicillin-sensitive have lost the pFLP2 plasmid and were verified by Sanger sequencing.

### Intracellular zinc quantification by ICP-MS

Pre-cultures in MS medium were inoculated from fresh MS agar plates and grown overnight at 37°C under agitation. MCM cultures were then inoculated at OD = 0.05 and incubated for 9 h in the same conditions. OD_600_ was measured before cells were harvested by centrifugation (4,000 × *g*, 20 min, 4°C). The pellets were washed twice with 1 mL of fresh MS + 1 mM EDTA followed by a wash with 1 mL of fresh MS. The pellets were dried overnight at 95°C. After acidic digestion, the metal quantification was determined by inductively coupled plasma mass spectrometry (ICP-MS) as described elsewhere (61).

### Pseudopaline and metal supplementation experiments

MCM cultures of the pseudopaline-deficient strain (PA14Δ*cntL*) used in planktonic and biofilm growths were supplemented with various externally added concentrations of chemical S-pseudopaline synthesized as described elsewhere (24) and resuspended in ultrapure water at 100 mM stock solution. For metal supplementation, MCM cultures of PA14Δ*cntL* were supplemented with ZnSO_4_, CuSO_4_, CoCl_2_, MnCl,_2_ or NiCl_2_ (10 mM stock solution, solubilized in ultrapure water) at 5 µM final.

### Co-cultures and estimation of relative fitness

Main MCM cultures (*n*= 3 per co-culture) were inoculated from independent overnight MS culture with an inoculum equivalent to OD_600_ of 0.05 per strain, and grown at 37°C with shaking at 180 rpm during 10h. At the endpoint, 2µL of culture was diluted in filtered PBS (0.22 µm pore size) containing 30 μg/mL chloramphenicol and measured on a BD FACS Fortessa flow cytometry system. Measurements were taken with a 561-nm laser excitation source/610-nm (20-nm bandpass filter) fluorescence acquisition channel in the case of mScarlet fluorescence, and an excitation/610-nm (20-nm bandpass filter) emission channel for msfGFP. Calibration of the experiments was performed as follows: forward and side scatter density plots were used to identify the bacterial cell population and exclude debris. Detection of the fluorescent populations was calibrated using non-fluorescent PA14 WT, as well as independent cultures from cells expressing mScarlet or msfGFP. A control experiment of co-culture mixing WT strains showed no fitness defect of individual reporters. All flow cytometry data were processed using FlowJo. To evaluate the proportion of each population (*n* = 100.000 per replicate), the number of msfGFP and mCherry events were counted and divided by the final number of events after gating.

### RNA sequencing and transcriptome analysis

RNA extraction for RNAseq was performed using the RNeasy Mini Kit (Qiagen) in combination with Qiashredder columns, as described previously (62). Briefly, main MCM cultures were inoculated with an initial OD_600_ of 0.05 from an overnight pre-culture in MS media and harvested after and directly mixed with an equal volume of RNA protect (Qiagen). RNA was extracted according to the manufacturer’s instruction with slight modifications. DNA removal was performed using the DNA-free Kit (Thermo Fisher Scientific, Waltham, MA, USA). RNA integrity was checked using the Agilent Bioanalyzer (RNA 6000 Nano Kit; Agilent). Ribosomal RNA and tmRNA were removed using a RNAse-H-digestion-based, organism-specific, depletion method (63). Sequencing of the samples was performed in paired-end mode (2 × 50 bp reads) on an Illumina NovaSeq 6000 device operated by the sequencing platform of the Helmholtz Center for Infection Research (GMAK). Sequencing reads were mapped to the PA14 reference genome using bowtie2 (64), and the number of reads per gene was assessed with FeatureCounts (65). Differential gene expression analysis between the PA14 WT and mutant strains was performed with the edgeR package (66) using the function glmTreat (fold-change 1.2). Genes were filtered using the edgeR function filterByExpr, and reads were normalized using the edgeR function calcNormFactors (trimmed mean of M values). Adjusted *P*-values were calculated for multiple tests using the Benjamini–Hochberg adjustment. The significance threshold was set to a false discovery rate (FDR) of ≤ 0.05 to identify differentially expressed genes. Functional enrichment analysis of Gene Ontology (GO) terms (67) was carried out using the hypergeometric test (R function phyper, adjusted *P*-value [FDR] < 0.05].

### RNA preparation and reverse transcription

RNAs were prepared from 25 mL culture of PA14 grown in LB or MCM after 10-h shaking at 37°C. The cells were harvested by centrifugation and frozen at −80°C. Total RNAs were isolated from the pellet using RNeasy mini Kit (Qiagen) according to the manufacturer’s instructions; and using TURBO DNAse (Invitrogen) to eliminate the contaminating DNA. The RNA quality was assessed by the TapeStation 4200 system (Agilent). RNA was quantified spectrophotometrically at 260 nm (NanoDrop 1000; Thermo Fisher Scientific). For cDNA synthesis, 1 µg total RNA and 0.5 μg random primers (Promega) were used with the GoScript Reverse Transcriptase (Promega) according to the manufacturer’s instruction.

### Quantitative real-time reverse transcriptional PCR (qRT-PCR)

qRT-PCR analyses were performed on a CFX96 Real-Time System (Bio-Rad, France). The reaction volume was 15 μL, and the final concentration of each primer was 0.5 μM. The cycling parameters of the qRT-PCR were 98°C for 2 min, followed by 45 cycles of 98°C for 5 s, 60°C for 10 s. A final melting curve from 65°C to 95°C was added to check the specificity of the amplification. To determine the amplification kinetics of each product, the fluorescence derived from the incorporation of EvaGreen into the double-stranded PCR products was measured at the end of each cycle using the SsoFast EvaGreen Supermix 2X Kit (Bio-Rad). The results were analyzed using Bio-Rad CFX Manager software, version 3.1 (Bio-Rad). The 16S RNA and *uvrD* genes were used as a reference for normalizations. For each point, a technical triplicate was performed. The amplification efficiencies for each primer pair were between 80% and 100%. All of the primer pairs used for qRT-PCR are shown in Table 2.

### Biofilm formation

Biofilm formation for quantitative evaluation was measured in clear, flat bottom, 24-well plates (Corning Costar 3526). Each overnight culture was inoculated at an OD_600_ of 0.2 in 1 mL fresh MCM and incubated at 30°C for 24 h. Pseudopaline complementation was performed with addition of 100 µM of synthetic S-pseudopaline to the medium before bacteria inoculation. Following incubation, planktonic bacteria and media were rinsed away with non-sterile deionized water. The wells were filled with 0.25% crystal violet solution, and after 15 min at room temperature, they were washed three times with deionized water. Any crystal violet staining on the bottom of the well was cleaned, and the plate was let to air-dry overnight at room temperature. Crystal violet rings were then solubilized in 30% glacial acetic acid. Adherent biofilm was quantified by measuring optical density of all samples at 550 nm and normalized to mean absorbance from wells containing WT strain. Images used for biofilm illustration are representative of three independent experiments. For analysis of CntO production by Western blot, biofilms were resuspended within the medium, and cells were centrifuged in order to resuspend the pellet in a volume of 1× Laemmli buffer, allowing a final cell concentration of 0.02 OD units/µL. CntO and housekeeping DsbA protein levels in the different protein samples were estimated by immunoblot experiments using anti-CntO or anti-DsbA antibodies.

Time-lapse qualitative biofilm formation was performed in flow chambers as previously described (68). Each experiment was repeated two times. The *P. aeruginosa* PA14 WT*::gfp* and PA14Δ*cntL::gfp* were tagged with green fluorescent protein (GFP), following the procedure described in reference 69, except that conjugation instead of electroporation was used to transfer plasmids into *P. aeruginosa*. Bacteria from MS overnight precultures were inoculated at OD_600_ of 0.1 in MCM in flow chambers with individual channels (dimensions of 1 × 4 × 40 mm^3^) and incubated for 3 h at 30°C before flow start. At day 11, tobramycin at 20 µg/mL was added and incubated for 24 h with stopped flow. At day 12, the flow was turned on for 30 min, before addition of propidium iodide and incubation for 30 min with stopped flow. The flow was started again for 24 h before observation at day 13. Observation was performed after 4, 7, 10, and 13 days with an Olympus FV-1000 microscope equipped with detectors and a filter set for monitoring GFP and propidium iodide. Z-stack images were used to reconstruct the 3D architecture of the biofilms, which were then analyzed using Fiji (70). Quantitative parameters, such as biovolume (µm³/µm²), average thickness, and surface coverage, were extracted. To account for potential differences in cell density, the number of cells per imaging field was estimated from GFP fluorescence intensity, allowing normalization of biofilm biovolume to cell number.

### Western blot analysis

For analysis of CntO production, protein samples from bacteria corresponding to 1 OD unit were resuspended in 50 µl 1× SDS-PAGE loading Laemmli buffer containing β-mercaptoethanol, heated at 95°C for 10 min, and 5 µL was loaded and separated on 12% acrylamide SDS-PAGE. After separation, the proteins were transferred onto nitrocellulose membranes for Western blot analysis. Transfers were performed with a semi-dry technique using Power Blotter Station (Invitrogen). The membranes were washed briefly in Tris-buffered saline, 0.10% Tween 20 (TBS-T) and then blocked in TBS-T + 10% milk for 1 h at RT. The membranes were incubated for 1 h at RT in TBS-T + 10% milk containing the primary antibodies (laboratory collection) directed against CntO or DsbA at 1/1,000 dilution where the housekeeping protein DsbA is used as loading control. This was followed by four washes in TBS-T before an incubation of 45 min at RT in TBS-T + 10% milk containing the secondary antibody anti-mouse or anti-rabbit-HRP conjugated at 1/5,000. Finally, after five more washes with TBS-T, the peroxidase reaction was revealed by chemiluminescence (SuperSignal West Dura Extended Duration Substrate, Thermo Scientific) and scanned with ChemiDoc Touch Imaging System (BioRad).

### Macrophage infections

#### Phagocytic uptake and survival

RAW264.7 (ATCC number: TIB-71TM) cells were routinely grown in DMEM (4.5 g/L glucose) and supplemented with 10% FCS, 20 mM HEPES, and 1% BSA at 37°C, 5% CO_2_. All cell cultures used in this study were tested and confirmed as mycoplasma-free. Invasion assays were performed as described previously (40). Briefly, 5 × 10^5^ RAW 264.7 cells were seeded in DMEM in a 24-well plate for phagocytic uptake or intracellular replication measurements. Before the start of the experiments, the supernatant of each well was discarded and replaced with 400 μL of fresh DMEM without phenol red. Eukaryotic cells were infected at a MOI of 1 with selected *P. aeruginosa* strains grown 10 h in MCM, washed with PBS, and adjusted to a final volume of 100 μL in fresh DMEM. The number of CFU in the inoculum dose is verified by serial plating on LB plates. Immediately after the start of the infection, a centrifugation step at 1,200 × *g* for 5 min was used to ensure cell-to-cell contact. To kill extracellular bacteria, 1 hpi, 100 μL of DMEM containing gentamicin was added to the final concentration of 50 μg/mL. At 2 hpi, cells were washed with PBS and lysed with PBS containing 0.5% (v/v) Triton X-100. CFUs were determined by plating of serial dilutions and compared to the parental strains. These CFUs represent the number of cells phagocyted and normalized to the inoculum. A replicate plate was used for survival assay, using the same inoculum and cells batch. After 6 h, cells were washed with PBS and lysed with PBS containing 0.5% (v/v) Triton X-100. CFUs were determined by plating of serial dilutions and comparedwith the parental strains. Intracellular survival is evaluated by the ratio of CFU recovered after 6 h and normalized to the number of cells phagocyted after 2 h.

#### Adherence assay

Adherence assay was performed as described before (40). Briefly, RAW234.7 cells were seeded in DMEM as detailed in the phagocytic uptake and survival assays and infected by *P. aeruginosa* strain grown in MCM for 10 h. Then, 10 µM of Cytochalasin D was added to cell cultures 2 h prior to infection to block phagocytosis. Directly after infection, the bacteria were centrifuged at 1,200 × *g* for 5 min to ensure cell-to-cell contact. At 2 hpi, the supernatant was removed. The cells were then gently washed twice with PBS and lysed using PBS containing 0.5% (v/v) Triton X-100. Serial dilutions were used to determine the adherent bacteria by plating and counting CFUs.

#### Cytotoxicity

A total of 50 μL of supernatant of infected RAW264.7 cells was collected 6 hpi, and LDH levels were measured using the CytoTox 96 Non-radioactive Cytotoxicity Assay (Promega) according to the manufacturer’s protocol. PBS serves as a negative control, and lysis solution of the manufacturer was used as a killing control. Values are expressed as % of the value obtained with the PA14 WT strain infection.

#### Measure of cytokine production

Macrophage supernatants were sampled 6 hpi. The TNF-α and IL-6 ELISA Max Standard Kit (Biolegend) was used to determine the TNF-α and IL-6 levels according to the manufacturer’s manual. Three different biological replicates were analyzed in technical triplicates, and a PBS-treated group served as negative control.

### Pulmonary mouse infections

#### Mice

Adult male C57BL/6jrj (B6) mice (6–8 weeks old) were obtained from Janvier (Le Genest Saint-Isle, France). All mice were housed under specific-pathogen-free conditions at the “PST Animaleries” animal facility (Université de Tours, France) and had access to food and water *ad libitum*.

#### *P. aeruginosa* infections

MCM precultures of WT and Δ*cnt* strains were used in this study. Mice were anesthetized with isoflurane 4%, and an operating otoscope fit with intubation specula was introduced to both maintain tongue retraction and visualize the glottis. A fiber optic wire threaded through a 20 G catheter and connected to a torch stylet (Harvard Apparatus, France) was inserted into the mouse trachea. Correct intubation was confirmed using lung inflation bulb test and 40 µL of the bacterial inoculum (2–8 × 10^6^ CFU) was applied using an ultrafine pipette tip. Inoculum size for infections was confirmed after each experiment by counting CFU on *Pseudomonas* isolation agar plates. Mortality, body weight, and clinical score of animals were monitored daily. The clinical score was calculated by recording the following symptoms: ruffled fur, hunched posture, motor impairment, breathing impairment, closed eyes, purulent eyes, and allocating one point to each. In all experiments, moribund animals—animals with a weight loss of more than 20% or clinical score of 7—were sacrificed for ethical reasons and considered as dead due to the infection.

#### Bronchoalveolar lavage, organ sampling, and bacterial load assay

At day 2 after infection, mice were sacrificed with intraperitoneal injection of pentobarbital. BAL was collected after *P. aeruginosa* infection by introducing a catheter into the trachea under deep pentobarbital anesthesia and washing sequentially the lung with 2 × 1 mL of 1× PBS at room temperature. The lavage fluid was centrifuged at 400 × *g* for 10 min at 4°C, and the supernatant was stored at −20°C for ELISA analysis. Lungs were perfused with 10 mL of PBS 1× and harvested in GentleMACS M tubes (Miltenyi Biotec, Germany) containing 2 mL of 1× PBS for microbiology assay. Bacterial load in lung homogenates or BAL (before centrifugation) was determined by plating tenfold serial dilutions on *Pseudomonas* Isolation Agar (PIA) plates, which were incubated at 37°C in a 5% CO_2_ atmosphere, and the CFUs were counted after 24 h.

#### Measure of cytokine production

The concentration of IL-6 and TNF-α in BAL supernatants and lung homogenates was measured using specific ELISA Max Standard Kit (Biolegend) according to the manufacturer’s manual.

### Statistics

Statistics was determined by analysis of variance (ANOVA) followed by a Tukey’s or Sidak’s post-test (respectively for Fig. 1, 2, 3A, C through F, 4D through F and Fig. 3B, 4A and B). Log-rank test was used for survival analysis (Fig. 4C). Figures and statistics were performed using GraphPad Prism 10.4.1.

## Data Availability

RNAseq data have been deposited in the Gene Expression Omnibus (GEO) database with the accession number GSE310904.

## SUPPLEMENTAL MATERIAL

Supplemental figures iai.00453-25-s0001.pdf Fig. S1 to S7.

Table S1 iai.00453-25-s0002.csv Pseudopaline-dependent transcriptome in MCM.

ASM does not own the copyrights to Supplemental Material that may be linked to, or accessed through, an article. The authors have granted ASM a non-exclusive, world-wide license to publish the Supplemental Material files. Please contact the corresponding author directly for reuse.
