# Supplementary material for: Pseudopaline-mediated zinc uptake by Pseudomonas aeruginosa drives clinically relevant phenotypes and infection outcomes
Source: Infect Immun. 2026 Jan 14;94(2):e00453-25. doi: 10.1128/iai.00453-25 (PMC12890022; doi:10.1128/iai.00453-25)
Supplement: Supplemental figures — Fig. S1 to S7. [file iai.00453-25-s0001.pdf]

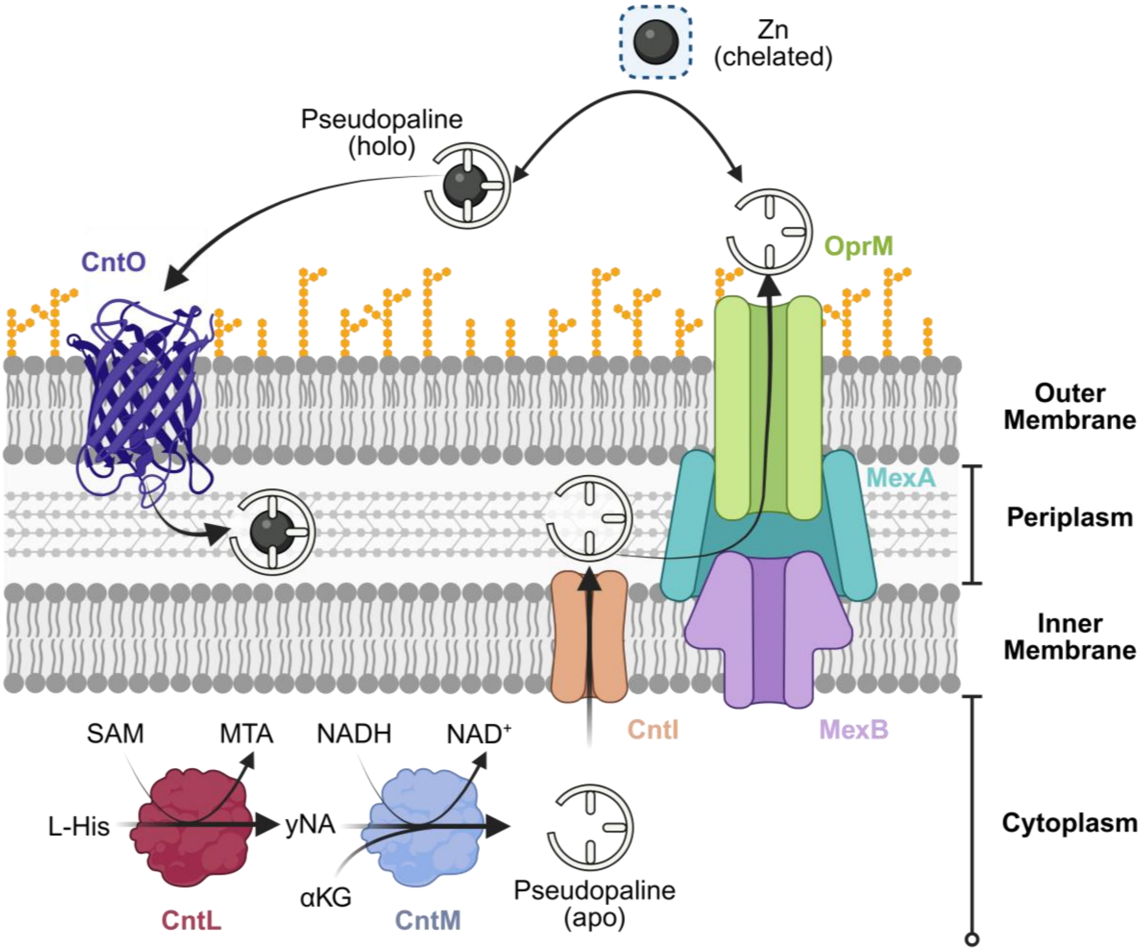

FIGURE S1: Summary of known steps of pseudopaline's biosynthesis and trafficking.
Pseudopaline is first synthesized in the cytoplasm in a two-step process by CntL and CntM. In the first step, CntL produces the intermediate yNA using S-adenosylmethionine (SAM) and L-histidine as substrates which exhibits chemical homology with xNA in staphylopine biosynthesis and nicotianamine (NA). The reaction releases 5'-methylthioadenosine (MTA) as a byproduct. This is followed by a reductive condensation of the yNA intermediate with a molecule of α-ketoglutarate (α-KG), catalyzed by CntM, to produce pseudopaline (1, 2). The metallophore is then secreted in the milieu through the bacterial envelope by the CntI inner membrane exporter and through the outer membrane by the efflux pump MexAB-OprM. In the milieu, pseudopaline can displace Zn from chelators such as EDTA or the calprotectine produced by the host, forming holo-pseudopaline. The latter is finally recovered by the bacterium through its specific outer membrane receptor, CntO. Insofar, the fate of pseudopaline after import in the periplasm remains unknown, and could involve a dedicated ABC importer as suggested by the genetic organization observed for other "opine-type" metallophores found in γ proteobacteria (3).

SUPPLEMENTARY REFERENCES

1. Lhospice S, Gomez NO, Ouerdane L, Brutesco C, Ghssein G, Hajjar C, Liratni A, Wang S, Richaud P, Bleves S, Ball G, Borezee-Durant E, Lobinski R, Pignol D, Arnoux P, Voulhoux R. 2017. *Pseudomonas aeruginosa* zinc uptake in chelating environment is primarily mediated by the metallophore pseudopaline. Sci Rep 7:17132.
2. McFarlane JS, Lamb AL. 2017. Biosynthesis of an Opine Metallophore by *Pseudomonas aeruginosa*. Biochemistry 56:5967-5971.
3. Laffont C, Arnoux P. 2020. The ancient roots of nicotianamine: diversity, role, regulation and evolution of nicotianamine-like metallophores. Metallomics 12:1480-1493.

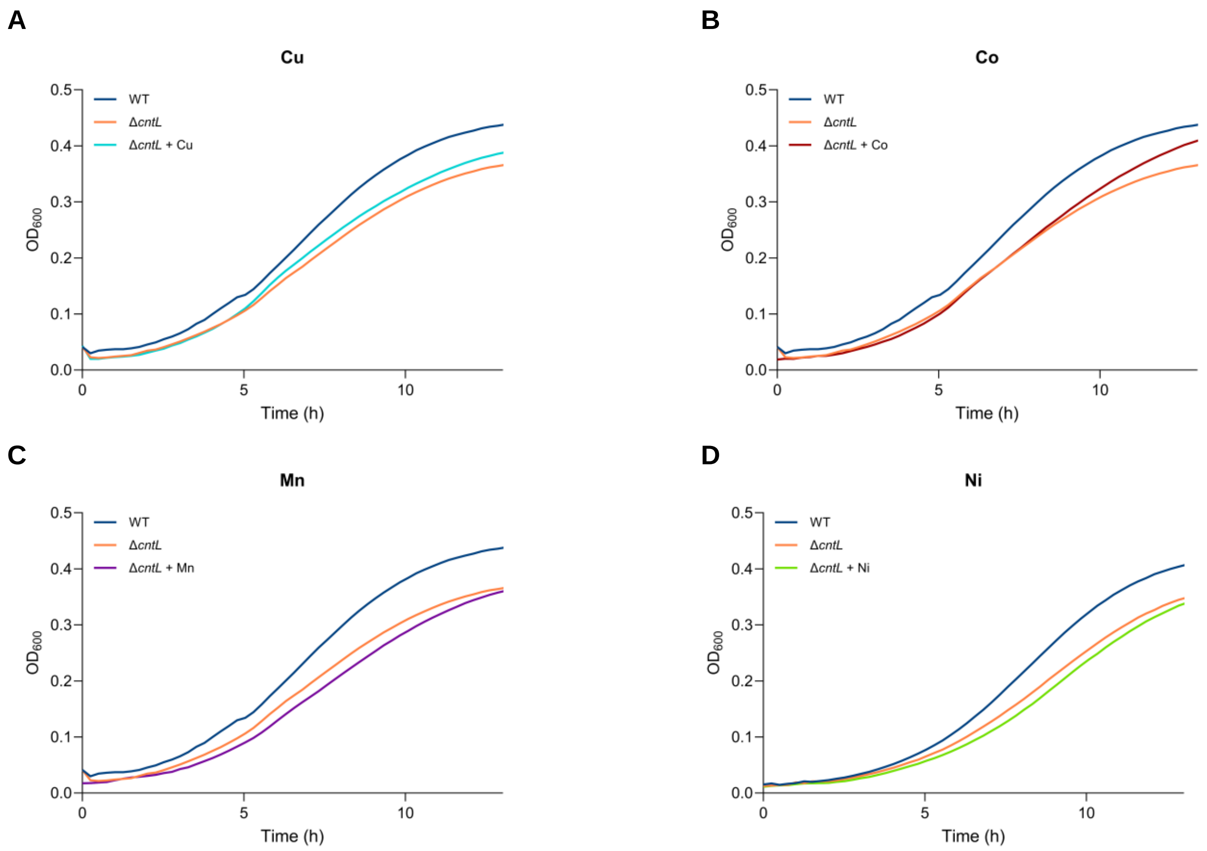

FIGURE S2: The growth of Δ*cntL* is not restored by the addition of other external metals

Impact of externally added metals on cell growth of PA14 wild-type (WT) and Δ*cntL* strains, supplemented or not with 5 µM of exogenous copper (Cu), cobalt (Co), manganese (Mn) or nickel (Ni) and grown in MCM.

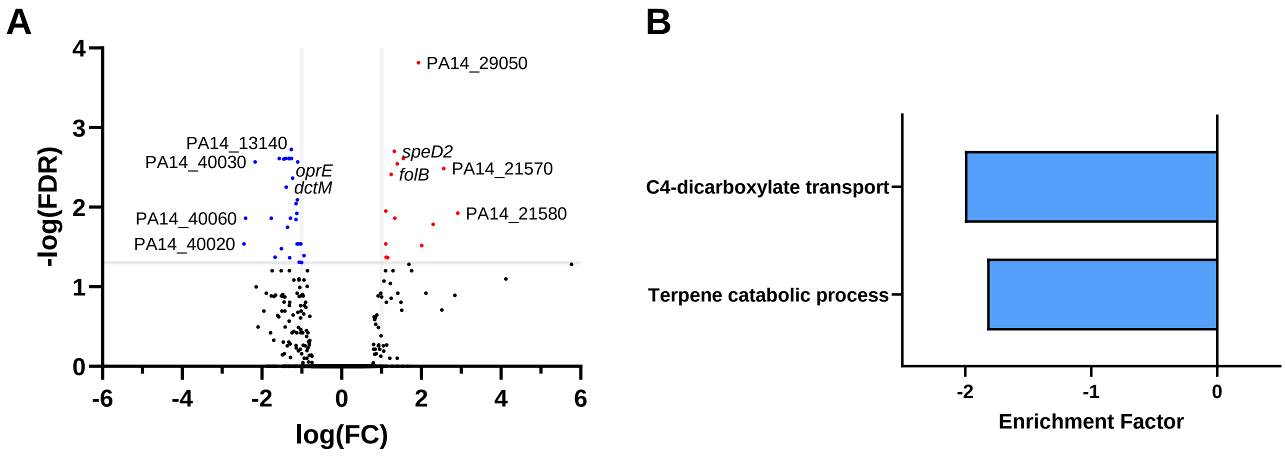

FIGURE S3: Pseudopaline-dependent transcriptome in MCM.

(**A**) Volcano plot depicting fold changes in mRNA levels comparing the Δ*cntL* mutant to PA14 WT (n = 3) grown in MCM. Red dots represent transcripts that are significantly upregulated [−log10(FDR) > 1.3, log2FC > 1], while blue dots represent transcripts that are significantly downregulated [−log10(FDR) > 1.3, log2FC < 1]. (**B**) Functional enrichment analysis of differentially expressed genes assigned to GO categories (hypergeometric test, FDR ≤ 0.05).

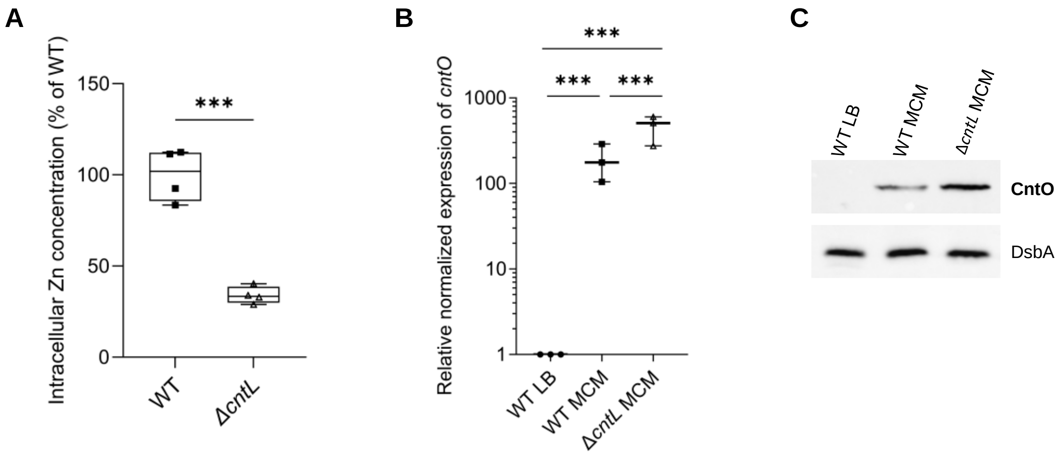

FIGURE S4: Direct correlation between intracellular zinc concentration and *cntO* gene expression and CntO protein detection.

(**A**) Intracellular zinc concentration and (**B**) *cntO* gene expression of PA14 wild-type (WT) and Δ*cntL* strains grown in MCM or LB medium. (**C**) For all panels, CntO and housekeeping DsbA protein levels in the different protein samples were estimated by immunoblot experiments using anti-CntO or anti-DsbA antibodies. Error bars, mean ± standard deviation (sd) of at least three independent biological replicates. *** Correspond to $p < 0.001$. (one-way ANOVA, Tukey post-hoc test).

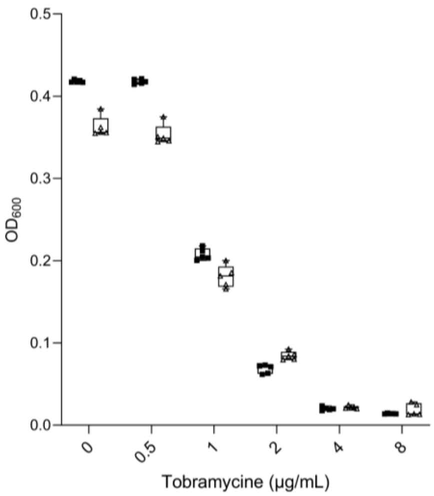

FIGURE S5: The deletion of *cntL* does not impact the tobramycin sensitivity of PA14 in planktonic growth in MCM.

Cell growth in MCM of PA14 wild-type (■) and Δ*cntL* (Δ) strains grown in presence of increasing concentrations of tobramycine. Error bars, mean ± standard deviation (sd) of at least three independent replicates.

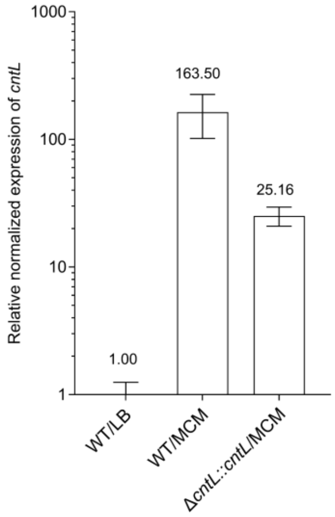

FIGURE S6: *cntL* expression is significantly reduced in the PA14 Δ*cntL::cntL* complemented strain.
cntL gene expression quantified by qRT-PCR of WT or Δ*cntL::cntL* strains grown in MCM or LB medium. Error bars, mean ± standard deviation (sd) of at least three independent replicates.

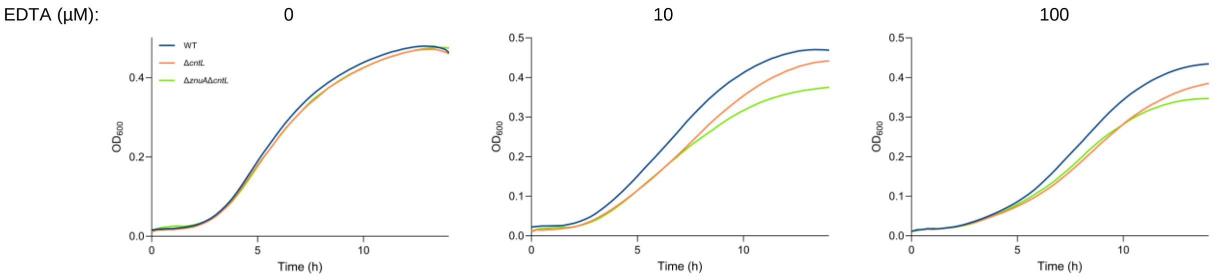

FIGURE S7: The Znu and Cnt zinc import pathways are hierarchically required under low, moderate and high zinc scarcity.

Planktonic cell growth of PA14 wild-type (WT), Δ*cntL*, and Δ*cntL*/Δ*znuA* strains in MS medium supplemented or not (0) by 10 (10) or 100 (100) µM of EDTA.

TABLE S1: Pseudopaline-dependent transcriptome in MCM. For each transcript are indicated the fold change (logFC), *p* value and adjusted *p* value (False discovery rate, FDR) used for generating the volcano plot presented in Supplementary Figure S2A.

See Excel file
